# Size-Dependent Performance of Abnormal-Focused U-Net Segmentation for Mammographic Lesion Detection: A Two-Stage Hybrid Training Approach

**DOI:** 10.7759/cureus.105360

**Published:** 2026-03-17

**Authors:** Kian A Huang, Haris K Choudhary, Hailey Mangio, Ahmad Alkadri, Hailey R Kramer, Neelesh S Prakash

**Affiliations:** 1 Radiology, University of South Florida Morsani College of Medicine, Tampa, USA

**Keywords:** artificial intelligence, breast cancer, computer-aided diagnosis, deep learning, image segmentation, lesion detection, mammography, u-net

## Abstract

Introduction

Breast cancer screening mammography faces challenges from variable radiologist performance and missed cancers. Deep learning segmentation models offer promise for automated lesion detection, but most training datasets are biased toward normal cases, limiting performance on clinically relevant abnormalities.

Methods

A custom-enhanced U-Net architecture was trained on annotated abnormal mammograms from the Digital Mammography Dataset for Breast Cancer Diagnosis Research (DMID). Training employed a two-stage approach: (1) patch-based pretraining on 224 × 224 lesion-centered crops with 2:1 negative-to-positive sampling, and (2) full-image fine-tuning with 35% hybrid patch sampling to preserve small-lesion sensitivity. A composite loss function combining focal loss and Tversky loss addressed class imbalance and boundary precision. Images were downsampled from native 4000-6000 pixels to 224 × 224 with Contrast Limited Adaptive Histogram Equalization (CLAHE) contrast enhancement. Performance was evaluated on 55 test images using Dice coefficient, Intersection-over-Union (IoU), pixel accuracy, Hausdorff distance, and lesion detection rate (IoU > 0.10), with size-stratified analysis across small (<500 pixels), medium (500-1500 pixels), and large (>1500 pixels) lesion categories.

Results

The model achieved a mean Dice score of 0.5793 (median, 0.7120), a mean IoU of 0.4902 (median, 0.5541), a pixel accuracy of 0.9930, and an overall lesion detection rate of 77.8% (43/55). Size-stratified analysis revealed pronounced performance gradients: small lesions demonstrated a 73.0% detection rate (27/37) with a mean Dice score of 0.698; medium lesions achieved an 84.6% detection rate (11/13) with a mean Dice score of 0.724; and large lesions showed a 100% detection rate (5/5) with a mean Dice score of 0.908. Among detected lesions, weak positive correlations were observed between lesion size and segmentation quality (Dice, r = 0.233; IoU, r = 0.215). The primary failure mode was missed detection of small lesions (n = 12; mean size, 253.3 pixels vs. 1137.6 pixels for detected lesions; p < 0.001), likely attributable to information loss during 18-27-fold resolution reduction.

Conclusions

Abnormal-focused U-Net training achieved strong segmentation for large lesions (Dice 0.908, 100% detection) but exhibited critical limitations for small abnormalities (27% miss rate), representing a significant clinical barrier given that early-stage cancers are primary screening targets. The resolution bottleneck from downsampling high-resolution mammography represents a fundamental architectural limitation. Unknown false-positive rates on normal mammograms - absent from training - preclude clinical deployment. Future work should prioritize multi-scale architectures, hybrid training incorporating normal cases, external validation across diverse datasets, and prospective evaluation in screening workflows before clinical translation.

## Introduction

In 2025, an estimated 316,950 cases of invasive breast cancer will be diagnosed among US women, with 42,170 deaths expected [1]. Breast cancer is the most commonly diagnosed cancer and the second leading cause of cancer death among women worldwide [2]. Approximately one in eight women in the US will be diagnosed with invasive breast cancer in their lifetime, and one in 43 will die from the disease [2]. Breast cancer incidence has been increasing by approximately 1% annually during 2012-2021, yet despite rising incidence, breast cancer death rates have declined 41% since 1990 due to improved screening, diagnosis, and treatment [2]. Screening mammography has been shown to decrease breast cancer mortality, with digital breast tomosynthesis recommended when available to further improve cancer detection and decrease callback rates [1,2]. Early detection enables identification of tumors when they are smaller and more likely to be node-negative, thereby reducing treatment morbidity and improving outcomes [3]. Despite the proven mortality benefits of mammography, interpretation faces substantial challenges stemming from inherent difficulty and subjectivity. Radiologists miss 10-30% of cancers despite most missed cancers being visible on the images, reflecting human perceptual and decision-making errors rather than technical limitations alone [4]. Interpretive performance varies considerably among radiologists, with mean screening performance showing sensitivity of 86.9% and specificity of 88.9% [5]. Along with this, the high prevalence of burnout among breast radiologists directly exacerbates the substantial challenges in mammographic interpretation by impairing diagnostic accuracy and performance [6]. The combination of human perceptual limitations, subjective interpretation, and burnout-related performance degradation underscores the need for technological solutions to both improve diagnostic accuracy and reduce radiologist cognitive load.

The emergence of artificial intelligence (AI) has provided an opportunity for clinicians to reduce the limitations associated with imaging-based cancer diagnosis. Deep learning (DL) is a particularly promising subtype of AI that can extract intricate information from large datasets. DL algorithms have been shown to exceed human performance in visual tasks, including the advanced analysis of medical imaging [7]. Convolutional Neural Networks (CNN), a class of DL algorithms, have already demonstrated high efficacy in detecting, segmenting, and classifying tumors across a wide range of imaging modalities [7,8]. Segmentation models, such as U-Net, offer promise in automating analysis to assist radiologists in reading mammography [8]. Segmentation is especially effective because it allows for models to work at the pixel level, detecting where abnormalities are specifically located in a given image [8]. This is significant in the context of breast cancer diagnosis because it provides decisional support to radiologists, giving them additional context in cases that may be difficult to assess alone, such as a patient with dense breasts [8]. Ultimately, these advancements demonstrate the promise of DL for the future of not only breast cancer diagnosis but imaging-based tumor diagnosis altogether, prompting further exploration into their application in clinical practice.

Many public mammography datasets are predominantly composed of normal or benign cases or biased toward specific clinical-use cases, resulting in data imbalance and limited diversity of abnormal presentations [9,10]. These dataset limitations contribute to well-described failures of DL models to generalize to complex, real-world abnormal cases [9,10]. Such constraints introduce representation bias, where a model fits well to its training data but fails to maintain performance when tested on images acquired in different clinical environments [11]. For instance, Wang et al. reported that multiple DL models achieving area under the receiver operating characteristic curves (AUROCs) between 0.71 and 0.79 on their internal validation set fell to 0.44 to 0.65 when evaluated on external mammography datasets with different populations and data distributions [9]. More recent studies reinforce this generalization gap: a high-performing ensemble DL model demonstrated reduced performance when applied to a more diverse screening cohort, and external validation showed that three of four state-of-the-art models performed poorly on publicly available datasets, with the best-performing model exhibiting substantial variability across datasets [12,13]. In contrast, Wang et al. demonstrated that a U-Net-based radiomics-DL fusion model trained exclusively on BI-RADS 4 abnormalities achieved strong benign-malignant classification performance on an external BI-RADS 4 validation cohort from a separate institution, suggesting that abnormal-focused training may mitigate some generalization limitations [14]. Collectively, the current literature highlights how massive data imbalance in public mammography datasets, combined with the heterogeneity of real-world abnormalities, constrains DL models trained on these datasets from learning the nuanced imaging characteristics necessary for reliable abnormality detection.

Although U-Net and its derivatives have shown good promise for biomedical image segmentation, their performance still remains imperfect due to several limitations. One of the most well-known problems in medical segmentation is class imbalance, where the lesion (foreground) occupies only a tiny fraction of the image while the vast majority is healthy tissue (background) [15]. This can be especially prevalent in mammography, where small breast lesions (often less than 15 mm) can account for less than 2% of the image pixels, which can lead to a severe class imbalance [15]. As a result, vanilla U-Net models tend to “over-learn” the background, because predicting background for most pixels minimizes the loss more easily [15]. In practice, this bias can lead to false negatives or missed detections, since the network effectively finds it easier to ignore the tiny lesion regions [15]. In order to address class imbalance, models such as SE-Attention U-Net incorporate attention mechanisms and hybrid, imbalance-aware loss functions that can amplify the contribution of small lesion pixels and penalize missed detections more heavily, which improves sensitivity to subtle or small breast masses [15].

U-Net also faces limitations in preserving fine spatial detail and accurately delineating small or irregular masses, as boundary and shape inaccuracies, particularly in mammography, where masses often have low-contrast margins and highly variable shapes and sizes [16]. These issues are considered major challenges for reliable CNN-based segmentation [16]. Additionally, U-Net can produce false positives by misclassifying background or non‑lesion structures (e.g., pectoral muscle, fibroglandular tissue, fat) as lesion or foreground tissue [17]. For example, in a study on fully automatic breast MRI segmentation of fibroglandular tissue, a conventional U-Net was reported to incorrectly label parts of the pectoral muscle and fat as fibroglandular tissue in low-density breasts [17]. Similarly, in a mammography-based soft-tissue lesion detection study, a U-Net-based approach reported an average of nearly eight false positives per image at high sensitivity, highlighting the risk of non-lesion regions being identified as potential lesions [18].

The primary objective of this work is to develop and evaluate a specialized diagnostic U-Net segmentation pipeline by training exclusively on annotated abnormal mammograms. We hypothesize that an abnormal-focused training paradigm, which prioritizes the features of confirmed lesions over traditional balanced datasets containing high proportions of normal tissue, will improve the segmentation and detection of complex, real-world abnormalities. Our approach utilizes a two-stage hybrid training strategy - combining lesion-centered patch pretraining with full-image fine-tuning - and integrates focal and Tversky loss functions to address class imbalance and boundary precision. Through size-stratified analysis, we systematically characterize size-dependent detection thresholds and failure modes, including resolution-dependent performance degradation. By providing a transparent assessment of these technical limitations, this study establishes a methodological framework for abnormality-focused medical image segmentation to inform future model development.

## Materials and methods

Dataset and preprocessing

Digital mammography images featuring annotated breast lesions were sourced from the Figshare repository via the Digital Mammography Dataset for Breast Cancer Diagnosis Research (DMID) [19]. This dataset provides high-resolution imagery accompanied by binary ground truth segmentation masks that delineate radiographically confirmed lesions. To optimize model training and prevent the segmentation algorithm from developing a bias toward negative predictions, the inclusion criteria were restricted to cases with available masks; normal or unannotated images were excluded from the final training set.

Custom DL models and all statistical calculations were implemented using the PyCharm Integrated Development Environment (IDE) (JetBrains s.r.o., Prague, Czech Republic) in a Python 3.10 environment (Python Software Foundation, Wilmington, DE). The neural networks were constructed and trained using the TensorFlow 2.15 framework (Google LLC, Mountain View, CA) with Keras API integration (Google LLC, Mountain View, CA).

Because the native clinical images ranged between approximately 4000 and 6000 pixels per side, intensity normalization and contrast enhancement were performed to mitigate contrast variability introduced during downsampling. First, images were converted to floating-point format and normalized to a 0-1 intensity range. Subsequently, Contrast Limited Adaptive Histogram Equalization (CLAHE) was applied (clip limit = 2.0; tile grid size = 8 × 8) to enhance local contrast while avoiding amplification of background noise. After preprocessing, masks were binarized using a threshold of 0.5 and reshaped to match the input dimensionality of the model.

Minor data augmentation was applied to increase robustness and reduce overfitting, including random rotation (0-10 degrees), horizontal flipping, elastic deformation, adaptive histogram equalization-based contrast enhancement, small random zoom (0-12%), and background-preserving cropping that ensured the lesion remained visible.

Dataset statistics, including positive pixel count and lesion-to-background ratio, were calculated to characterize segmentation sparsity. As expected, lesions represented a small proportion of total pixel volume, motivating architectural and sampling strategies to mitigate class imbalance.

Model architecture

A custom-enhanced U-Net architecture was implemented for segmentation. The network followed a symmetric encoder-decoder structure with skip connections to preserve spatial detail. The encoder consisted of three downsampling blocks using 3 × 3 convolutional layers, batch normalization, ReLU activation, and 2 × 2 max-pooling. The number of filters per block increased progressively (64, 128, 256), culminating in a 512-filter bottleneck layer. A dropout layer (rate = 0.3) was applied at each downsampling stage to reduce overfitting.

The decoder path used Conv2DTranspose layers for upsampling, concatenated with their corresponding encoder feature maps to recover fine-grained lesion boundaries. The output layer consisted of a 1-channel sigmoid-activated convolution, producing a probability map representing per-pixel lesion likelihood.

This enhanced configuration was selected to balance computational efficiency with sensitivity to small lesion structures, which are easily lost in deeper architectures when dealing with highly sparse masks.

Two-stage hybrid training strategy

The study initially identified 510 mammograms, from which 269 abnormal cases were selected for model development and evaluation. To ensure robust training and independent validation, the dataset was partitioned using a 60/20/20 split, resulting in a training cohort of 159 images, a validation cohort of 55 images, and a final independent test set of 55 images. Because many lesions occupied only a small fraction of the total breast area, a two-stage hybrid training framework was developed to first learn local lesion features and then integrate them into the global anatomical context.

Stage 1: patch-based pretraining

To bias early learning toward lesion features, model pretraining was performed on lesion-centered cropped patches of size 224 × 224 pixels. Bounding boxes were automatically generated from binary masks, and patches were extracted around lesion centroids with small random spatial perturbations to introduce positional variance. For each positive patch, additional background (negative) patches were sampled at a 2:1 negative-to-positive ratio, provided that the mask coverage was <5% to avoid inadvertent lesion inclusion.

Geometric augmentation (horizontal/vertical flipping) was applied stochastically during training. The patch-based dataset was split 80/20 for training and validation.

Stage 2: full-image fine-tuning with hybrid sampling

After patch pretraining, encoder weights were partially transferred to a full-image U-Net with identical architecture. The model was then fine-tuned on full-resolution preprocessed mammograms.

To prevent catastrophic forgetting of small-lesion representation, a custom hybrid data generator randomly replaced 35% of full-image samples with upsampled lesion patches, enforcing lesion presence in a subset of mini-batches. This allowed the model to learn lesion localization in context while retaining sensitivity to small abnormalities.

Both pretraining and fine-tuning were optimized using the Adaptive Moment Estimation (ADAM) - a commonly used adaptive learning rate algorithm that maintains individual learning rates for each parameter, utilizing the first and second moments of the gradients to ensure stable convergence in complex loss landscapes. optimizer (learning rate = 1e-4). Early stopping, learning-rate decay, and checkpoint saving were applied based on the validation Dice score.

To address the inherent class imbalance in mammographic data (where lesion pixels are sparse compared to background), a hybrid loss function was employed. This included focal loss, which applies a modulating factor to the cross-entropy loss to down-weight "easy" examples and focus training on "hard" misclassified samples. Additionally, we utilized Tversky loss, which allows for asymmetric weighting of false positives and false negatives. 

Evaluation metrics

Performance was evaluated on a held-out test set using several complementary segmentation metrics. The Dice similarity coefficient quantifies the overlap between predicted and ground-truth masks, emphasizing how well the model captures shared pixels. Intersection-over-Union (IoU) similarly measures overlap but penalizes mismatches more strictly by comparing the intersection of the two masks with their union. Pixel-level accuracy reflects the proportion of correctly classified pixels across the entire image, offering a global measure of correctness. The symmetric Hausdorff distance - computed only when both ground truth and predictions contained non-zero segmentation - captures boundary agreement by measuring the greatest distance between the edges of the predicted and true regions.

To assess lesion-level detection, we additionally defined a lesion detection rate, calculated as the proportion of images with IoU > 0.10. Detection rates were subsequently compared across lesion size categories using statistical tests described below. All predictions were binarized using a threshold of 0.5 prior to evaluation.

The Dice coefficient measures the harmonic mean of precision and recall for segmentation overlap, emphasizing the proportion of shared pixels between the predicted mask and ground-truth mask. IoU quantifies segmentation overlap by comparing the intersection of the predicted and true masks with their union, providing a stricter penalty for mismatched regions. Pixel-level accuracy represents the proportion of correctly classified pixels - both positive and negative - relative to all pixels in the image.

Statistical analysis

All statistical analyses were performed using Python (version 3.10) with the SciPy and StatsModels libraries. Descriptive statistics, including mean, median, and standard deviation, were calculated for segmentation performance metrics.

To evaluate whether segmentation performance varied according to lesion size, lesions were categorized into three predefined groups based on mask pixel area: small (<500 pixels), medium (500-1500 pixels), and large (>1500 pixels). Comparisons were performed using the Kruskal-Wallis test, a nonparametric alternative to one-way ANOVA that does not assume normal distribution of the data.

## Results

**Figure 1:**
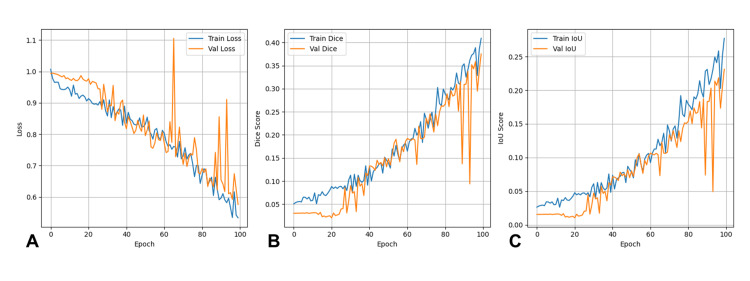
Model Training and Validation Performance Across Epochs Training and validation (val) curves demonstrating model convergence over 100 epochs. (A) Loss curves showing a steady decrease in both training (blue) and validation (orange) loss with minimal divergence, indicating effective learning without overfitting. (B) Dice coefficient progression showing consistent improvement from approximately 0.05 to 0.40, with training and validation metrics closely aligned. (C) Intersection-over-Union (IoU) score progression demonstrating a similar upward trajectory, reaching approximately 0.25 by epoch 100. The close proximity of training and validation metrics across all panels indicates successful generalization to unseen data.

Model training progressed without instability, and both the loss and segmentation performance curves demonstrated gradual improvement across epochs. As shown in Figure 1, the training loss steadily decreased throughout the training period, with the validation loss following a similar downward trend, albeit with expected fluctuations characteristic of small-sample medical imaging pipelines and patch-based learning. Correspondingly, the Dice and IoU performance curves demonstrated a consistent upward trajectory over time, ultimately reflecting convergence rather than overfitting, with close proximity between training and validation metrics near the end of training. These trends indicate progressive learning of relevant structural boundaries with maintained generalizability.

Quantitative evaluation of the final model on the independent test set (n = 55) showed a mean Dice coefficient of 0.5793 and a median Dice of 0.7120 (Table 1). The mean IoU was 0.4902 with a median IoU of 0.5541, and the mean pixel-level accuracy was 0.9930. The mean Hausdorff distance was 100.34 pixels, although instances with missing predicted segmentation masks resulted in undefined values. The model successfully detected lesions in 77.8% of cases (43/55) when applying the predefined detection criterion of IoU greater than 0.10. Visual qualitative assessment confirmed that in most true-positive cases, the model accurately localized and delineated the lesion, and probability heatmaps showed well-defined areas of high activation aligning with clinically relevant abnormal regions.

**Table 1:** Overall Model Performance on Test Set Summary statistics of key segmentation metrics (n = 55) evaluated on the independent test set. Dice coefficient and Intersection-over-Union (IoU) measure segmentation overlap accuracy, pixel accuracy reflects global classification performance, and Hausdorff distance quantifies boundary agreement between predicted and ground-truth masks. Hausdorff Distance was calculated excluding cases with missing masks (n = 12). Detection rate: 77.8% (43/55 lesions with IoU > 0.10).

Metric	Mean	Median	Standard Deviation
Dice Coefficient	0.579	0.712	0.344
IoU Score	0.490	0.554	0.333
Pixel Accuracy	0.993	0.996	0.008
Hausdorff Distance (pixels)	100.34	77.47	72.15

Size-stratified detection performance

Detection performance demonstrated a clear size-dependent pattern (Table 2). Missed detections (n = 12, 21.8%) occurred predominantly among smaller lesions, with an average lesion size of 253.3 pixels compared to 1137.6 pixels for successfully detected lesions (p < 0.001). When stratified by lesion size category, small lesions (<500 pixels) demonstrated a detection rate of 73.0% (27/37), medium lesions (500-1500 pixels) achieved 84.6% (11/13), and large lesions (>1500 pixels) were detected in 100% of cases (5/5). These findings demonstrate a strong size-dependent detection trend, suggesting that smaller abnormalities represent the primary challenge for the current segmentation pipeline.

**Table 2:** Detection and Segmentation Performance Stratified by Lesion Size Performance metrics across three lesion size categories (small: <500 pixels, medium: 500-1500 pixels, large: >1500 pixels) demonstrate size-dependent detection rates and segmentation quality. Detection rate represents the proportion of lesions successfully identified with Intersection-over-Union (IoU) > 0.10.

Size Category	Ground Truth Lesions (n)	Detected (n)	Missed (n)	Detection Rate (%)	Mean Lesion Size (pixels)
Small (<500 pixels)	37	27	10	73	223.4
Medium (500-1500 pixels)	13	11	2	84.6	843.5
Large (>1500 pixels)	5	5	0	100	5153.6
Overall	55	43	12	78.2	749.5

Size-stratified segmentation performance

Among successfully detected lesions (n = 43), segmentation performance also varied according to lesion size (Table 3). Small lesions demonstrated a mean Dice coefficient of 0.698 ± 0.242 and a mean IoU of 0.573 ± 0.235, while medium lesions achieved a Dice coefficient of 0.724 ± 0.189 and an IoU of 0.596 ± 0.182. Large lesions demonstrated substantially higher segmentation performance, with a mean Dice coefficient of 0.908 ± 0.056 and a mean IoU of 0.836 ± 0.072.

**Table 3:** Statistical Comparison of Segmentation Performance Across Lesion Size Categories Statistical comparison of segmentation performance across lesion size categories among successfully detected lesions (n = 43). Differences between groups were evaluated using the Kruskal-Wallis test. Intersection-over-Union (IoU) and SD (standard deviation). Overall mean: Dice = 0.5793, IoU = 0.4902 when including missed detections as zero.

Metric	Small	Medium	Large	Statistical Test
Dice coefficient ± SD	0.698 ± 0.242	0.724 ± 0.189	0.908 ± 0.056	Kruskal-Wallis (H = 14.09, p < 0.001)
IoU score ± SD	0.573 ± 0.235	0.596 ± 0.182	0.836 ± 0.072	Kruskal-Wallis (H = 13.92, p < 0.001)

To evaluate whether segmentation performance differed across lesion size groups, a Kruskal-Wallis test was performed. The analysis demonstrated a significant difference in Dice scores across lesion size categories (H = 14.09, p = 0.0009). A similar pattern was observed for IoU scores, with the Kruskal-Wallis test also indicating significant differences between groups (H = 13.92, p = 0.0010). These results indicate that segmentation performance varies significantly across lesion size categories, with larger lesions generally demonstrating higher overlap metrics.

These results indicate that once lesions surpass the detection threshold, larger abnormalities are segmented with substantially greater accuracy, likely due to improved feature representation and clearer boundary definition at the model's operating resolution.

Qualitative error analysis

**Figure 2:**
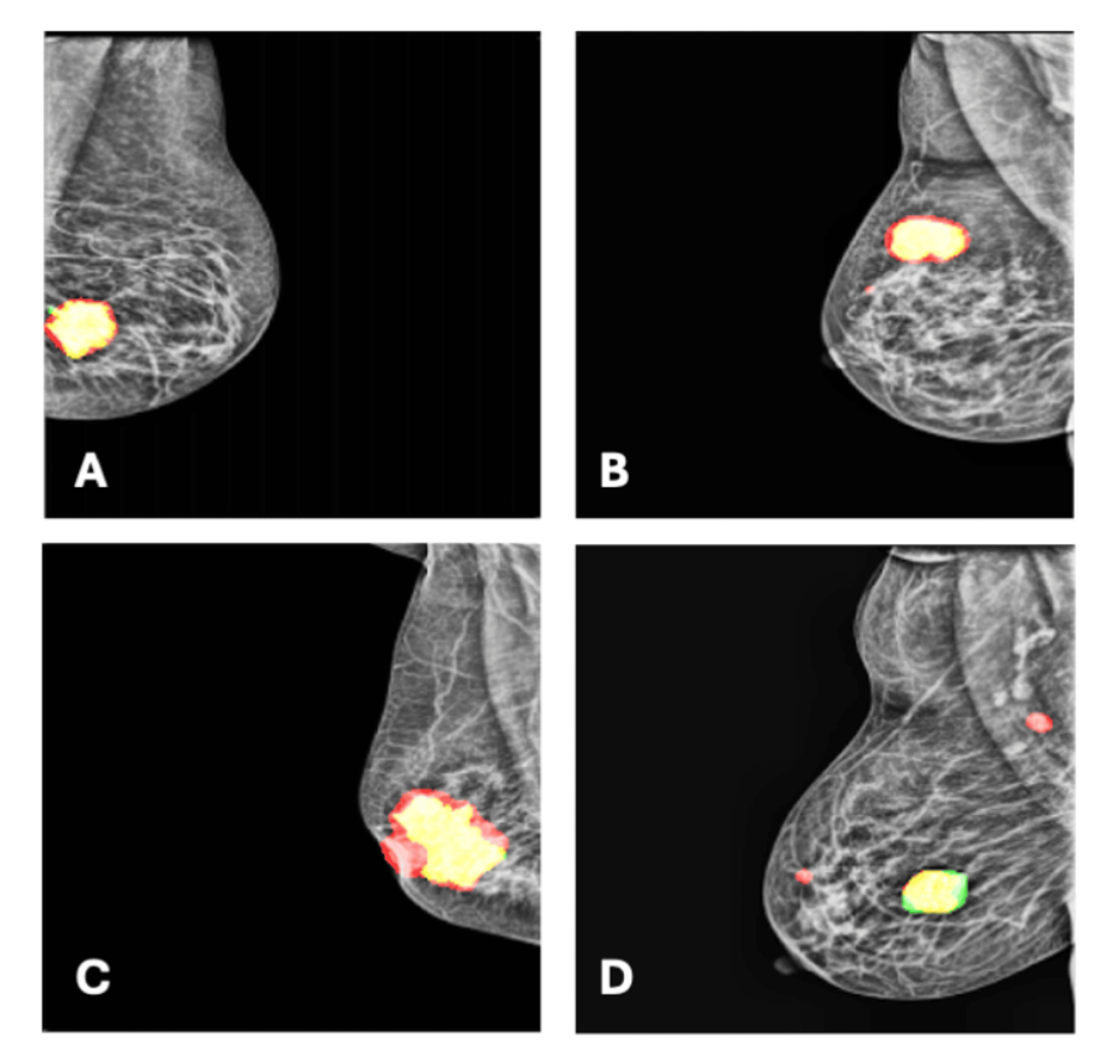
Representative Examples of Model Segmentation Performance on Mammographic Lesions Mammogram segmentation results showing predicted lesion boundaries (red) overlaid on ground truth annotations (green). (A, B) Large lesions with moderate segmentation accuracy, showing slight boundary discrepancies. (C) Large irregular lesion with good overall detection but minor over-segmentation extending beyond the ground truth boundary. (D) Example of segmentation challenges with underprediction of the largest lesion and multiple small lesions detected as false positives.

Representative examples of prediction behavior are shown in Figure 2, including input mammograms, ground-truth masks, probability maps, threshold masks, and fused overlays. These examples demonstrate several recurrent patterns encountered during evaluation. In many successful cases, the predicted masks closely approximated the size, location, and contour of the ground-truth annotations, particularly in lesions occupying a moderate to large proportion of the breast area. In a subset of images, the model slightly overestimated lesion extent, a pattern most frequently observed in structurally dense regions containing irregular parenchymal texture or peripheral glandular overlap. In contrast, under-segmentation occurred primarily in cases with low-contrast or irregularly shaped lesions, where the model detected only the more radiographically conspicuous core of the abnormality rather than its full annotated extent. False-positive predictions were observed in a minority of cases, often appearing as small isolated activations within dense tissue regions on the prediction heatmaps. False-negative events were strongly associated with small abnormalities that, after resizing from the original 4000-6000-pixel resolution to 224 × 224 pixels, occupied less than 0.5% of the total pixel area and were therefore at the resolution limit of the model. In several of these cases, the probability maps showed faint but sub-threshold activation at the lesion site, suggesting partial internal representation without confident segmentation. This pattern is consistent with the quantitative finding that 27% of small lesions (<500 pixels) were not detected, representing the primary failure mode of the current architecture.

## Discussion

This study developed a two-stage hybrid U-Net trained exclusively on abnormal mammograms for automated lesion segmentation. The model achieved a mean Dice coefficient of 0.5793 and a mean IoU of 0.4902, with successful lesion detection in 77.8% of cases. Stratified analysis revealed pronounced size-dependent performance: small lesions (<500 pixels) showed 73.0% detection, medium lesions 84.6%, and large lesions 100%. Among detected lesions, large abnormalities achieved mean Dice scores of 0.908 compared to 0.698 for small lesions. Statistical comparison using the Kruskal-Wallis test demonstrated significant differences in segmentation performance across lesion size categories (Dice: H = 14.09, p = 0.0009; IoU: H = 13.92, p = 0.0010). These findings highlight both the potential and fundamental limitations of applying standard U-Net architectures to high-resolution mammography.

Size-dependent performance and the resolution bottleneck

The 27% miss rate for small lesions represents the model's primary failure mode and stems from fundamental information loss during preprocessing. Native mammography images (4000-6000 pixels) required downsampling to 224 × 224 pixels - an 18-27-fold linear reduction. For a 250-pixel lesion at native resolution, the downsampled representation contains only 0.3-0.8 equivalent pixels, approaching theoretical detectability limits [15,18]. Even with contrast enhancement, critical diagnostic information is irretrievably lost at this scale. Faint sub-threshold activation in probability maps suggests the network formed partial representations of small abnormalities but lacked sufficient feature resolution for confident predictions, indicating the limitation is architectural rather than algorithmic [16,18].

The strong performance observed for large lesions, near-perfect detection, and Dice scores exceeding 0.90, suggests that the underlying U-Net architecture and training strategy are fundamentally effective when lesion features exist at compatible spatial scales [20]. This dichotomy suggests improvements should focus on multi-scale processing or higher-resolution inference rather than wholesale architectural redesign [16].

Among successfully detected lesions, segmentation performance varied significantly across lesion size categories according to Kruskal-Wallis testing. The significant test statistics for both Dice and IoU indicate that the distributions of segmentation accuracy differ between the lesion size groups rather than arising from a single common distribution. While this analysis does not identify which specific groups differ, the descriptive statistics suggest that segmentation accuracy tends to improve for larger lesions. This pattern likely reflects stronger feature representation and clearer boundary definition at the model’s operating resolution when lesions occupy a larger proportion of the input image. These results confirm that lesion size is a statistically significant factor influencing segmentation performance.

Abnormal-focused training: benefits and generalization concerns

Training exclusively on abnormal mammograms forced the network to learn discriminative features among pathologic findings rather than simply distinguishing normal from abnormal tissue. This approach yielded relatively strong segmentation quality for detected lesions despite modest dataset size, suggesting efficient learning of lesion-specific imaging features. This aligns with Wang et al. (2025), who demonstrated that DL trained exclusively on BI-RADS 4 abnormalities achieved robust external validation performance [14].

However, this paradigm introduces critical concerns. By never exposing the model to normal mammograms, we have no empirical data on false-positive rates in screening populations where most examinations are normal [10]. The model learned what lesions look like but never learned "definitively normal" breast parenchyma. This knowledge gap could manifest as elevated false-positive rates when encountering normal anatomical variants, prominent fibroglandular tissue, or benign findings [17,18]. Occasional false-positive predictions in dense tissue regions within our abnormal-only test set provide a concerning preview of this failure mode, similar to challenges reported for complex fibroglandular tissue segmentation in breast MRI [17].

These concerns are compounded by well-documented generalization failures in mammography DL [11,13]. Wang et al. demonstrated AUROC drops from 0.71-0.79 to 0.44-0.65 during external validation [9], while Velarde et al. showed three of four state-of-the-art models performed poorly on external datasets [13]. Our single-dataset, abnormal-only training likely further increases the risk of reduced generalization, consistent with prior reports of performance drops in external validation [9,13].

Future work should explore hybrid training strategies leveraging abnormal-focused pretraining while incorporating normal cases during fine-tuning. A curriculum learning approach, beginning with abnormal-only training to establish lesion features, followed by mixed training for normal-abnormal discrimination, culminating in normal-heavy training to reduce false positives, may offer an optimal balance supported by evidence that staged or progressive training can improve feature learning and segmentation performance [14,20]. 

Technical limitations and failure modes

Beyond size-dependent detection failures, qualitative analysis revealed specific patterns. Over-segmentation occurred most frequently in heterogeneous breast density, where the model extended boundaries into adjacent fibroglandular tissue [15,17], reflecting challenges in distinguishing lesion margins from textural variations in complex backgrounds [16]. Context-aware attention mechanisms or multi-scale feature aggregation could address this by considering broader anatomical context [15,20].

Under-segmentation occurred with low-contrast or irregularly shaped lesions, where only radiographically conspicuous cores were captured while subtle peripheral extensions were missed [16,18]. This reflects a fundamental tension: ground-truth annotations represent expert consensus, but boundaries of infiltrative masses may be inherently ambiguous [16,18]. The model's conservative learned boundaries reduce false positives but compromise complete lesion delineation [18].

False-positive predictions revealed specific anatomical confounders. The pectoral muscle occasionally triggered activation due to its well-defined boundary [17], and overlapping fibroglandular tissue sometimes produced predictions mimicking mass lesions [17,18]. These suggest the model learned generic "abnormal-looking" features without sufficient contextual understanding to exclude normal structures [16]. Solutions may include explicit anatomical masking, multi-view integration, or adversarial training with challenging normal cases [15].

The 27% miss rate for small lesions highlights a fundamental resolution bottleneck, as downsampling native 4000-6000 pixel images to 224 × 224 - an 18-to-27-fold linear reduction - destroys high-frequency features necessary for early-stage detection. This information loss resulted in a mean Hausdorff distance of 100.34 pixels, indicating that boundary errors occasionally spanned nearly half the image width due to poor peripheral delineation and isolated false activations. These findings confirm that standard downsampling is insufficient for clinical screening, reframing the current model’s utility as diagnostic decision support for conspicuous abnormalities rather than a standalone detector. To bridge this gap, future work must prioritize multi-scale architectures, such as Feature Pyramid Networks, or native-resolution patch-based inference to preserve the critical spatial detail required for comprehensive sensitivity.

Clinical translation: capabilities and critical gaps

The model's higher segmentation performance for larger lesions suggests potential value as a confirmatory diagnostic tool, highlighting obvious abnormalities with interpretable probability maps [18]. However, the 27% small-lesion miss rate is unacceptable for clinical applications requiring comprehensive sensitivity [4]. Early-stage cancers - offering the best prognosis and the primary target of screening - are precisely the cases most likely to be missed. Given these limitations, deployment as a standalone screening tool would be inadvisable, consistent with prior discussions on generalization risks in mammography AI [3,11].

A more realistic near-term deployment might position the model as a triage or diagnostic tool identifying high-confidence large lesions for immediate review while allowing low-suspicion cases to follow standard workflow [4,11]. This leverages the model's strength (reliable large-lesion detection) while minimizing risk from its weakness, as all cases would still receive human interpretation [4].

Future directions

Addressing identified limitations requires coordinated advances. The resolution bottleneck demands multi-scale architectures like Feature Pyramid Networks processing images at multiple resolutions simultaneously, or patch-based inference at full resolution with intelligent patch selection [16,20]. Attention mechanisms and transformer-based architectures (TransUNet, Swin-UNet) could model long-range dependencies and selectively focus on relevant regions while suppressing false activations [15,20].

Training methodology should employ progressive curriculum learning: abnormal-focused pretraining to establish lesion features, fine-tuning on full abnormal images for contextual localization, mixed training for diagnostic discrimination, and normal-heavy training with hard negative mining to reduce false positives [14]. Larger, more diverse datasets with explicit small-lesion oversampling and multi-institutional sources spanning diverse populations and equipment would enable more robust generalization [10,11,13].

Evaluation frameworks warrant reconsideration. Lesion-level detection metrics assessing presence and approximate location may better reflect clinical utility than pixel-level segmentation metrics [16,18]. Size-stratified reporting should become standard, as aggregate metrics obscure limitations in clinically important subgroups [15,16]. External validation across multiple independent datasets and prospective validation in actual screening and diagnostic workflows are essential before claims of clinical readiness [9,12,13].

Study limitations

Several limitations constrain the interpretation of these results. Most notably, the DMID dataset lacks patient demographic information (age, race, ethnicity) and clinical metadata (breast density, pathology), preventing an analysis of algorithmic bias or performance variance across different patient subgroups. The absence of demographic and clinical metadata is a significant limitation, as background breast density, a primary confounder for AI-based segmentation, varies substantially across different demographic groups [21]. This lack of granularity prevents an assessment of whether the model maintains consistent performance across diverse populations, representing a critical gap in establishing true algorithmic safety prior to clinical deployment. Consequently, the model’s performance on specific populations remains unknown, and further validation on diverse, well-characterized clinical datasets is required before clinical translation.

Additionally, single-dataset evaluation limits generalization assessment across diverse clinical environments and populations [9,13]. The absence of normal mammograms in training and testing prevents assessment of false-positive rates-arguably the most clinically critical metric [10]. The relatively small test set (n = 55) reduces statistical power. We did not compare against other state-of-the-art architectures (transformer-based models) or evaluate downstream classification performance [14]. Ground-truth annotations may contain imperfections, as lesion boundaries are often ambiguous and subject to inter-rater variability [18].

## Conclusions

This study demonstrates that a two-stage hybrid U-Net model trained exclusively on abnormal mammograms can achieve strong segmentation performance for large breast lesions (mean Dice 0.908, 100% detection rate) but exhibits critical limitations for small lesions, with a 27% miss rate for lesions under 500 pixels. The size-dependent performance pattern reflects a fundamental resolution bottleneck, as downsampling native 4000-6000 pixel mammograms to 224 × 224 pixels results in substantial information loss that disproportionately affects small early-stage cancers. While the abnormal-focused training paradigm successfully enabled efficient learning of pathologic features, the complete absence of normal mammograms during training introduces unmeasured false-positive risks that preclude clinical deployment without further validation.

Future work must address these limitations through multi-scale architectures capable of processing high-resolution images, hybrid training strategies that incorporate both normal and abnormal cases to reduce false positives, and rigorous external validation across diverse clinical populations and equipment. The path to clinical translation requires not only technical innovation but also transparent assessment of failure modes and realistic appraisal of where AI can meaningfully augment radiologist performance. This work establishes a methodological framework for abnormality-focused segmentation while clearly delineating the substantial validation work required before such approaches can be responsibly deployed in breast cancer diagnostic workflows.
